# Arbuscular Mycorrhizal Fungi Can Compensate for the Loss of Indigenous Microbial Communities to Support the Growth of Liquorice (*Glycyrrhiza uralensis* Fisch.)

**DOI:** 10.3390/plants9010007

**Published:** 2019-12-19

**Authors:** Meng Yu, Wei Xie, Xin Zhang, Shubin Zhang, Youshan Wang, Zhipeng Hao, Baodong Chen

**Affiliations:** 1 State Key Laboratory of Urban and Regional Ecology, Research Center for Eco-Environmental Sciences, Chinese Academy of Sciences, Beijing 100085, China; carfeild@163.com (M.Y.); xieweibisheng@yeah.net (W.X.); xinzhang@rcees.ac.cn (X.Z.); bdchen@rcees.ac.cn (B.C.); 2 University of Chinese Academy of Sciences, Beijing 100049, China; 3 Institute of Plant Nutrition and Resource, Beijing Academy of Agriculture and Forest Science, Beijing 100097, China; zbinb@163.com (S.Z.); wangyoushan5150@163.com (Y.W.)

**Keywords:** arbuscular mycorrhizal fungi, indigenous microorganisms, liquorice, secondary metabolites, soil sterilization

## Abstract

Soil microorganisms play important roles in nutrient mobilization and uptake of mineral nutrition in plants. Agricultural management, such as soil sterilization, can have adverse effects on plant growth because of the elimination of indigenous microorganisms. Arbuscular mycorrhizal (AM) fungi are one of the most important beneficial soil microorganisms for plant growth. However, whether AM fungi can compensate for the loss of indigenous microbial communities to support plant growth and metabolism is largely unknown. In this study, a pot experiment was conducted to investigate the effects of AM fungi on plant growth and secondary metabolism in sterilized and unsterilized soil. We used liquorice (*Glycyrrhiza uralensis* Fisch.), an important medicinal plant as the host, which was inoculated with the AM fungus *Rhizophagus irregularis* or not and grown in unsterilized or sterilized soil. Plant photosynthesis traits, plant growth and nutrition level, concentrations of the secondary metabolites, and expression levels of biosynthesis genes were determined. The results showed that soil sterilization decreased plant growth, photosynthesis, and glycyrrhizin and liquiritin accumulation, and moreover, downregulated the expression of related biosynthesis genes. Inoculation with *R. irregularis* in sterilized soil offset the loss of indigenous microbial communities, resulting in plant growth and glycyrrhizin and liquiritin concentrations similar to those of plants grown in unsterilized soil. Thus, AM fungi could compensate for the loss of indigenous microbial communities by soil sterilization to support plant growth and secondary metabolism.

## 1. Introduction

Numerous microorganisms exist locally in soil and live harmoniously with plants, and these microorganisms are actively involved in organic decomposition, nutrient cycling, and plant health [1]. Among the beneficial soil microorganisms, arbuscular mycorrhizal (AM) fungi are obligate symbiotic fungi that can form symbiotic associations with most terrestrial plants [2]. These fungi have attracted increasing attention because of their positive role in stimulating plant growth and plant adaptation to various environmental stresses, such as nutrient deficiency and pathogen/herbivore attack [3,4,5]. In mycorrhizal symbiosis, a plant allocates a portion of carbohydrate to AM fungi to support their growth. In return, AM fungi help the plant uptake of water and mineral nutrients from soil, especially the nutrients with poor mobility, such as phosphorus (P) [6,7]. In addition, AM symbiosis can regulate the biosynthesis and accumulation of secondary metabolites to protect plants against abiotic and biotic stress [8,9]. 

The overuse of chemical fertilizers, pesticides, and herbicides in agricultural production systems and unreasonable cropping systems can destroy the structure and function of soil microbial communities [10]. The high cost of agricultural production and underlying adverse environmental effects have led to the development of sustainable strategies to enhance plant productivity without damage to the environment [11]. One countermeasure is to reintroduce beneficial microorganisms as bioinoculants to facilitate nutrient uptake and strengthen plant resistance to biotic and abiotic stresses, instead of overusing fertilizers and pesticides in agriculture [12]. However, given the complex plant−Microorganism and microorganism−Microorganism interactions, and the high dependence of these interactions on environmental conditions, these partnerships are difficult to optimize to promote plant growth and productivity. As the key soil microbial group, AM fungi can serve not only as a biofertilizer, but also organize the assembly of plant-associated beneficial microbiomes within the host root and rhizosphere soil [13]. For example, Banerjee et al. [14,15] used high-throughput sequencing associated with network analysis to reveal that AM fungi are highly connected taxa that individually or in a group exert a considerable influence on soil microbiome, and their removal could cause a dramatic shift of the community structure and functioning of the whole root or rhizosphere soil microbiome. Whether AM fungi can compensate for the loss of soil indigenous microorganisms to support plant growth and metabolism is largely uncertain.

Liquorice (*Glycyrrhiza uralensis* Fisch.) has been used as a popular medicine for more than a thousand years for multiple medical purposes [16,17]. This plant belongs to the perennial herb of the Fabaceae family, which is widely grown in arid and semi-arid areas worldwide. In the natural environment, liquorice roots are colonized by several types of microbes, including actinomycetes, diverse rhizobia, and other soil bacteria, such as *Pseudomonas*, as well as AM fungi with plant growth-promoting activity [18,19,20,21,22]. Wang [19] investigated the distribution of AM fungi in liquorice roots and rhizosphere soils in Inner Mongolia, China, and found that liquorice roots are intensively colonized by AM fungi. Moreover, a variety of AM species, including *Acaulospora*, *Diversispora*, *Scutellospora*, *Funneliformis*, *Rhizophagus*, and *Glomus*, could be isolated from liquorice rhizosphere soil. These indigenous AM fungi may play important roles in helping liquorice roots take up nutrients and protect plants against other biotic stresses to promote liquorice growth [19]. Unfortunately, direct evidence supporting the relationship between these AM fungi and plant growth performance remains scarce. Lekberg and Koide [23] conducted a meta-analysis concerning the relation between the abundance of AM fungi and general plant performance and found that increased colonization results in a yield increase of approximately 23% across all management practices. This finding reveals the relation between AM fungi and plant growth and indicate the important roles of AM fungi in soil microbes. AM fungi also play important roles in facilitating glycyrrhizin and liquiritin accumulation. These substances are the main active ingredients of liquorice roots and rhizomes with a wide range of pharmacological effects and, thus, have great economic value [16,24]. The concentrations of glycyrrhizin and liquiritin in liquorice plants are considered the most important indices for evaluating the quality of liquorice products [25]. Our previous studies demonstrated that AM symbiosis could upregulate glycyrrhizin and liquiritin biosynthesis genes, such as the farnesyl diphosphate synthase gene (*FPS*), β-amyrin synthase gene (*β-AS*), cytochrome P450 monooxygenase 88D6 gene (*CYP88D6*), 72A154 gene (*CYP72A154*), lupeol synthase gene (*LUS*), and chalcone synthase gene (*CHS*), to promote glycyrrhizin and liquiritin accumulation [16,26,27,28,29,30].

In the present study, we hypothesized that AM fungi can efficiently compensate for the loss of indigenous soil microbial communities to maintain plant growth. We performed a greenhouse pot experiment with liquorice inoculated with/without AM fungus *Rhizophagus irregularis* Walker & Schuessler (BGC AH01) (+M/−M) and grown in sterilized/unsterilized soils (S/US). We chose *R. irregularis* as the test AM fungus due to the following reasons. (1) *Rhizophagus* has a global distribution and could be successfully adapted to different environment conditions [31,32,33,34]. (2) Our previous studies verified that *R. irregularis* shows excellent promoting effects on liquorice growth and active ingredient accumulation in sterilized soil [28,30]. However, the importance of the roles of *R. irregularis* in promoting plant performance relative to that of soil indigenous microbes remains largely unclear. Plant biomass, photosynthetic rate, leaf chlorophyll concentrations, and root nutrient concentrations were determined to evaluate plant growth. The concentrations of glycyrrhizin and liquiritin and the expression levels of related biosynthesis genes were detected to assess the quality of the products of liquorice plants. 

## 2. Results

### 2.1. Mycorrhizal Colonization

No mycorrhizal colonization was observed in the −M roots under sterilized conditions (Table 1). The mycorrhizal colonization rate (M%) and arbuscule abundance (A%) in the +M roots significantly (*p* < 0.01) increased compared to −M roots regardless of soil sterilization. Similarly, no significant differences in M% and A% were observed in +M plants between the sterilized and unsterilized treatments. The interactive effects of sterilization and AM inoculation treatment on M% and A% were significant.

### 2.2. Plant Growth Parameters

Soil sterilization and AM inoculation markedly affected plant height, shoot, and root biomass (Table 2). Significant interactions of AM inoculation with soil sterilization were found. In unsterilized soils, the −M and +M plants showed no significant differences in plant height and DW. In sterilized soils, AM inoculation significantly increased plant height and DW. In general, the +M plants in sterilized soils showed similar plant height and DW compared with plants grown in unsterilized soils, irrespective of AM inoculation conditions.

### 2.3. Chlorophyll Concentration, Net Photosynthetic Rate, and Stomatal Conductance

**Figure 1:**
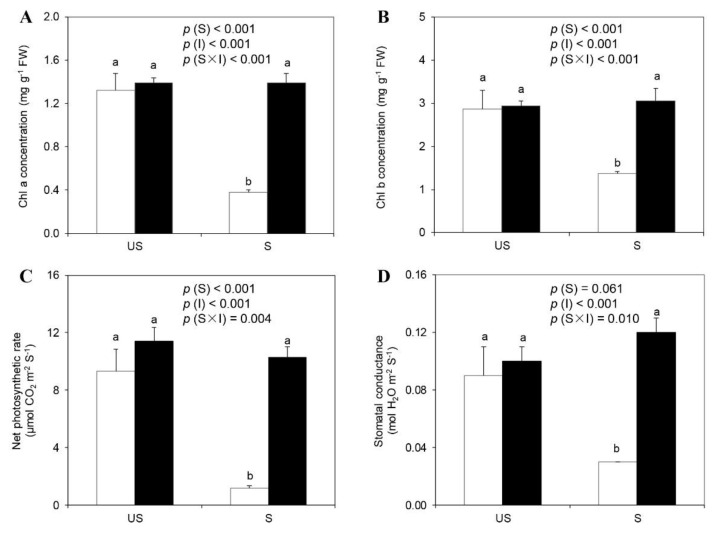
Chlorophyll a (**A**) and chlorophyll b (**B**) concentrations, net photosynthetic rate (**C**) and stomatal conductance (**D**) of liquorice plants under different treatments (□ non-inoculation; ■ AM inoculation). US and S represent unsterilization and sterilization treatments, respectively. Data are presented as means ± standard error (SE; *n* = 4). Treatment effects (*S*: Sterilization; *I*: Inoculation) are tested by two-way ANOVA and are shown as *p* values in the figure. Different lowercase letters above the columns indicate a significant difference between corresponding treatments at *p* < 0.05 by Tukey’s HSD test.

Soil sterilization negatively affected leaf chlorophyll concentration (Figure 1A,B). As a result, the Chlorophyll (Chl) a and Chl b concentrations of the −M plants in sterilized soils were markedly lower than those in unsterilized soils. In sterilized soils, the +M plants exhibited higher Chl a and Chl b concentrations compared with the −M plants. No significant differences were found in Chl a and Chl b among the +M plants in sterilized soils and the −M or +M plants in unsterilized soils.

Soil sterilization reduced leaf net photosynthetic rate (Pn) and stomatal conductance (Cond), whereas AM inoculation significantly improved plant photosynthesis traits (Figure 1C,D). Significant interactions of soil sterilization and AM inoculation were found for Pn and Cond. In unsterilized soils, Pn and Cond showed no significant differences between the −M and +M plants, while AM inoculation in sterilized soils markedly increased Pn and Cond by 7.6-fold and 3.5-fold, respectively, compared with the −M plants. No significant differences were observed among the +M plants in sterilized soils and −M or +M plants in unsterilized soils. 

### 2.4. Plant Nutrient Concentrations

In −M plants, soil sterilization significantly decreased C and P in the shoot and P and K in the root, but increased N, K, Ca, and Mg in the shoot and C, N, and Mg in the root (Table 3). As expected, no significant differences in C, N, P, K, Ca, and Mg in the shoot and root were observed between +M plants in sterilized soils and −M or +M plants in unsterilized soils. In unsterilized soils, no significant differences in the concentrations of C, N, P, K, Ca, and Mg were found between −M and +M plants, except for a decrease in Ca in +M plant shoots compared with that in the −M plants. In sterilized soils, AM inoculation markedly increased C and P in the shoot and P and K in the root but decreased N, K, Ca, and Mg in the shoot and C, N, and Mg in the root (Table 3).

For the −M plants, soil sterilization significantly decreased C:N ratios in the shoots and roots but increased C:P and N:P ratios (Table 3). In sterilized soil, AM inoculation increased the shoot and root C:N ratios but decreased the C:P and N:P ratios. Moreover, the AM plants in sterilized soils showed similar values of C:N:P ratios to the −M or +M plants in unsterilized soils. 

### 2.5. Root Glycyrrhizin, Liquiritin Concentrations, and Yields

**Figure 2:**
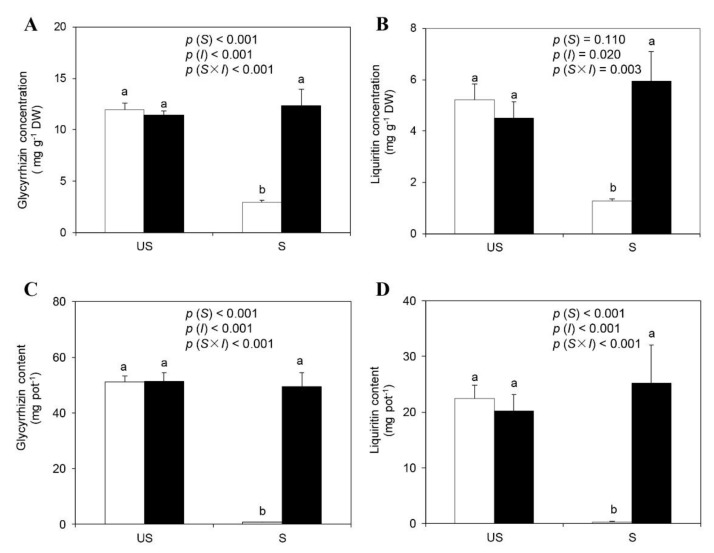
Liquorice root glycyrrhizin (**A**) and liquiritin (**B**) concentrations and glycyrrhizin (**C**) and liquiritin (**D**) contents under different treatments (□ non-inoculation; ■ AM inoculation). US and S represent unsterilization and sterilization treatment, respectively. Data are presented as means ± SE (*n* = 4). Treatment effects (*S*: Sterilization; *I*: Inoculation) are tested by two-way ANOVA and are shown as *p* values in the figure. Different lowercase letters above the columns indicate a significant difference between corresponding treatments at *p* < 0.05 by Tukey’s HSD test.

Soil sterilization significantly decreased glycyrrhizin and liquiritin concentrations in the roots of the −M plants by 76% and 75%, respectively, whereas no significant differences were detected between the +M plants in sterilized and unsterilized soils (Figure 2A,B). Conversely, *R. irregularis* inoculation significantly increased glycyrrhizin and liquiritin concentrations in the liquorice roots in sterilized soils. No significant differences were observed among the +M plants in sterilized soils and −M or +M plants in unsterilized soils for glycyrrhizin and liquiritin in the roots. Similarly, the yields (concentrations × root DWs) of glycyrrhizin and liquiritin significantly increased after AM inoculation of sterilized soil but decreased after soil sterilization in comparison with those of −M plants (Figure 2C,D). 

### 2.6. Expression of Genes for the Biosynthesis of Glycyrrhizin and Liquiritin

**Figure 3:**
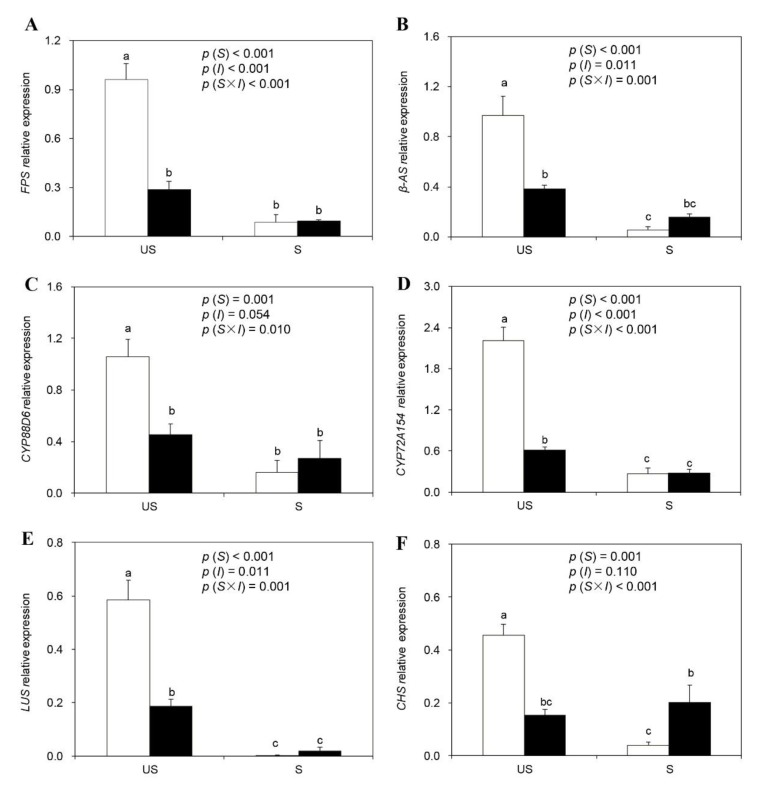
Relative gene expression of *FPS* (**A**), *β-AS* (**B**), *CYP88D6* (**C**), *CYP72A154* (**D**), *LUS* (**E**), and *CHS* (**F**) in roots of liquorice plants under different treatments (□ non-inoculation; ■ AM inoculation). US and S represent unsterilized and sterilization treatment, respectively. Data are presented as means ± SE (*n* = 4). Treatment effects (*S*: Sterilization; *I*: Inoculation) are tested by two-way ANOVA and are shown as *p* values in the figure. Different lowercase letters above the columns indicate a significant difference between corresponding treatments at *p* < 0.05 by Tukey’s HSD test.

Significant interactions were observed between soil sterilization and AM inoculation treatments for all gene expression levels (Figure 3). Soil sterilization significantly downregulated the expression of the genes involved in the biosynthesis of glycyrrhizin and liquiritin, including *FPS*, *β-AS*, *CYP88D6*, *CYP72A154*, *LUS*, and *CHS* irrespective of AM inoculation. AM inoculation significantly downregulated the expression of *FPS*, *β-AS*, *CYP88D6*, *CYP72A154*, *LUS*, and *CHS* under unsterilized conditions. By contrast, no significant differences were observed between the −M and +M plants under sterilization conditions, except for a significant upregulation of *CHS* by AM inoculation. No significant differences in gene expression were detected in the +M plants between the unsterilized and sterilization treatments, except for the downregulation in the expression of *CYP72A154* and *LUS* by soil sterilization. 

## 3. Discussion

Plants largely rely on soil microorganisms to utilize soil nutrients and maintain health [14,35]. Several studies have underlined the positive effects of indigenous soil microbes on plant growth and agricultural productivity [15,36]. While the role of microbial communities in ecosystem functioning is unequal, there exist some specific microbial group, such as AM fungi, being key drivers of many ecosystem processes, including soil nutrient cycling, water uptake and plant growth [14,15]. Our previous studies have proven that AM fungi play important roles in promoting plant growth [28,30], however, the relative importance and effectiveness of indigenous soil microbial communities and the key beneficial microorganisms, such as AM fungi, are rarely considered [37]. In this study, we used liquorice as the host plant to test the hypotheses that soil indigenous microorganisms are indispensable for plant growth, and that AM fungi may compensate for the loss of indigenous microbial communities to support plant growth. As proposed, our results indicated that AM fungi could take the role of indigenous microbes in terms of promoting plant growth as well as the accumulation of glycyrrhizin and liquiritin under sterilized conditions. 

Indigenous microorganisms in rhizosphere soil play a vital role in plant growth and agricultural sustainability [38]. Beneficial microorganisms, such as plant-growth promoting rhizobacteria (PGPR) and mycorrhiza fungi, can directly facilitate plant growth by assisting nutrient and water acquisition, or regulating plant hormone metabolisms, as well as by indirectly defending plants against pathogens [39]. For example, the rhizobium *Bradyrhizobium* sp., and *Mesorhizobium* sp., and the AM fungi of genus *Paraglomus* in the rhizosphere of liquorice are involved in N fixation and P uptake [40]. Hao et al. [41] proved that inoculation with the rhizobium *M. tianshanense* and the AM fungus *R. irregularis* enhances liquorice growth under different water regimes. However, modern agricultural managements usually harm the indigenous microorganisms. Excessive amounts of fertilizers, intensive tillage, and overuse of pesticides, herbicides, and fungicides can dramatically reduce the quantity and diversity of indigenous microorganisms in soil [31]. The loss of microbial diversity in soil can damage a series of soil ecosystem services [42]. For example, the application of pesticide to soil decreases rhizobium activity and root colonization by AM fungi, which ultimately inhibits the growth of several bean species, namely *Vigna sinensis*, *Phaseolus vulgaris*, and *Lupinus albus* [43]. In this study, we sterilized soil by radiation to eliminate indigenous microorganisms, including AM fungi and other beneficial microbes. Without soil microorganisms, the growth traits of liquorice, including plant leaf chlorophyll contents, photosynthetic efficiency, P concentrations, and plant dry weight, were significantly decreased/inhibited compared with that of plants grown in unsterilized soil, suggesting the important roles of indigenous soil microorganisms in supporting plant growth. 

In addition to the important roles of soil microorganisms in supporting plant growth, soil microorganisms play a role in regulating plant secondary metabolisms [9,44]. In *Arabidopsis*, *Pseudomonas fluorescens* SS101 can trigger defense responses and promote the biosynthesis of camalexing and glucosinolates [45]. AM fungi can regulate plant secondary metabolites, such as terpenoids and flavonoids [46,47,48]. In this study, plants grown in sterilized soil showed significant reductions in glycyrrhizin and liquiritin concentrations, indicating the importance of soil microorganisms in plant secondary metabolism. Nutrient level, especially those of P, essentially affects the accumulation of glycyrrhizin and liquiritin through direct involvement in the biosynthesis of secondary metabolites, regulation of C:N:P ratios and expression of the related genes [30]. Our results indicated that the non-AM plants grown in unsterilized soil had higher levels of root P and C:N ratios and higher concentrations of glycyrrhizin and liquiritin compared with those in sterilized soil. Consistent with the pattern for the accumulation of glycyrrhizin and liquiritin, soil sterilization also significantly downregulated the expression of genes related to the biosynthesis of glycyrrhizin and liquiritin. 

Interestingly, although soil sterilization has been speculated to increase nutrient availability due to changes in the physical structure of soil and death of soil microbes, which results in cells lysis and nutrient release [49], inhibition of soil pathogens as a process of soil sterilization could also affect the microbiological properties of sterilized soil [50] and, ultimately, promote plant growth. For example, Miransari et al. [49] found that sterilization effectively increases plant P uptake (30–57% higher in sterilized soil than in unsterilized soil) and promotes corn’s (*Zea mays*) growth. In the present study, however, non-AM and AM plants cultivated in sterilized soil showed marked nutrient and growth inhibition and no significant effects, respectively, when compared with plants cultivated in non-sterilized soil regardless of the AM inoculation conditions, despite the fact that AM plant roots show a high AM colonization rate in sterilized soil. This result may be attributed to several reasons as follows. (1) Although the tested unsterilized soil has a large indigenous AM fungus, the amount of nutrients released by sterilization may still be too low for the needs of liquorice, which has been demonstrated to require a large supply of nutrients to grow well [51]. (2) Plant growth is affected not only by nutrient availability but also by other factors, such as phytohormones. For example, some bacteria produce substances that stimulate plant growth, such as the hormone auxin [52] While soil sterilization eliminates indigenous microbes, including some pathogens, it could also disrupt the possible beneficial effects of soil microbes to host plants. For example, soil sterilization has been demonstrated to have strong effects on the above- and below-ground traits of *O. europaea*, which were significantly decreased in sterilized soils [53].

AM symbiosis can improve plant mineral nutrition, especially P, and regulate the biosynthesis of secondary metabolites to enhance plant resistance [47]. In the current study, AM inoculation markedly improved plant growth and facilitated the accumulations of glycyrrhizin and liquiritin compared with the non-AM plants in sterilized soil, which has been proven in our previous studies [28,30]. However, we observed no significant difference in plant growth, including plant height, plant dry weights, nutrient concentrations, photosynthesis traits, and accumulations of glycyrrhizin and liquiritin between the AM plants in sterilized soil and non-AM plants in unsterilized soil, suggesting that AM symbiosis could take over the role of indigenous soil microbial communities to a certain extent to support plant growth and biosynthesis of secondary metabolites. The pronounced effects of AM inoculation on plant performance in this study could mainly be attributed to the improved nutrient uptake by AM symbiosis, especially P [47], as the soil used in this experiment was still depleted in P, although we supplemented P to the growth substrate before sowing. This deduction could be evidenced by the much higher N:P ratio of non-AM liquorice plants cultivated in sterilized soil (N:P = 57.1 in plant shoot and 43.4 in plant root) and lower N:P ratio of AM plants cultivated in sterilized soil (N:P = 12.7 in plant shoots and 6.87 in plant roots), as it was proposed that plant N:P ratio reflected plant nutrient conditions, and a high N:P ratio (N:P > 16) indicated that plants were suffering from severe P limitation [31,54]. 

Exogenous AM fungi can compete with indigenous microbial communities to produce different effects on plant growth [55]. Indigenous microorganisms, including native AM fungi, show great advantages in colonizing plant roots because of their priority in terms of allocating root space, resources, and immune responses of the host plants compared with colonizers; such priority may intensify the competition between exogenous AM fungi and indigenous microorganisms [37,56]. However, this competition also depends on the ecological environment. Under stressful conditions, such as low nutrient availability, plant responses to the competition between AM and other microorganisms may change, for example, from synergistic to antagonistic, and affect plant performance [57]. Miransari et al. [49] revealed that, when the soil is highly compacted, competition for soil resources among soil microorganisms, including AM fungi and other microbes, increases. In the present study, liquorice roots were colonized by indigenous mycorrhizal fungi, and inoculation with exogenous AM fungus significantly increased the root colonization intensity in unsterilized soils. However, no significant differences in plant growth and secondary metabolite concentrations were observed between −M and +M plants under unsterilized conditions. This result suggests the complex interactions of indigenous microorganisms (with native AM fungi) and exogenous AM fungi on plant performance. 

The non-responsiveness of plant growth to exogenous AM fungi has been observed in other studies. For example, Köhl and van der Heijden [58] investigated the effects of exogenous AM inoculation on the growth of *Trifolium pretense* and *Lolium multiflorum* in eight unsterilized soil samples. The results of these authors showed that AM inoculation does not influence the total N and P concentrations of plants in all soil types but the plant biomass response varies with soil type, although exogenous AM inoculation significantly increases total root colonization in all soil types. Verbruggen et al. [59] concluded that three main factors determine the effect of AM inoculation on plant performance: (1) competitiveness of native AM species to the introduced AM fungi because of the dominant role and adaptation of these fungi to the local environment, (2) plant and soil compatibility with the exogenous AM fungi, and (3) priority effects between native and introduced AM fungi. Although the non-AM and AM plants in our study exhibited similar plant growth rates under unsterilized conditions, the effects of exogenous and indigenous AM fungi and their interactions on plant growth could not be distinguished. 

In this study, we also detected the expressions of genes related to glycyrrhizin and liquiritin biosynthesis. Interestingly, AM inoculation significantly down-regulated the expression of plant biosynthesis genes in unsterilized soil. Moreover, this effect was more pronounced in plants cultivated in sterilized soil than in non-AM plants cultivated in unsterilized soil, although plants in all three treatments showed similar glycyrrhizin and liquiritin concentrations. In general, secondary metabolite biosynthesis in plants can be facilitated either by increasing precursors in the related biosynthetic pathways or by regulating the expression of metabolite synthase genes [47,60]. Improved photosynthetic efficiency and nutrient status, especially P, could provide substrates for secondary metabolite biosynthesis [30,46,47]. In this study, AM plants in sterilized and unsterilized soils showed chlorophyll concentrations, photosynthetic efficiency, and plant nutrients similar to those of non-AM plants cultivated in unsterilized soil, which may also provide similar substrates to the biosynthesis pathway of secondary metabolites and determine the accumulation of glycyrrhizin and liquiritin in liquorice plants. The carbon-nutrient balance hypothesis is assumed to explain the induction of secondary metabolites in plants [61]. According to this hypothesis, the availability of nutrients, including P, regulates the accumulation of carbon-based secondary metabolites, such as glycyrrhizin and liquiritin, and plants allocate more carbon to carbon-based secondary metabolites when plants have a higher C:N ratio [30,61]. This hypothesis may also be used to explain the different patterns of secondary metabolite accumulation and biosynthesis gene expression observed, as all plants, e.g., AM plants in sterilized and unsterilized soil and non-AM plants in unsterilized soil, showed similar root C:N ratios. However, the mechanisms underlying mismatches in gene expression and secondary metabolite accumulation require further assessment.

## 4. Materials and Methods

### 4.1. Experimental Materials

Seeds of *G. uralensis* were provided by China National Traditional Chinese Medicine Corporation, China. The selected seeds of uniform size and plump were dipped into 50% H_2_SO_4_ for 30 min and then surface-sterilized by 10% H_2_O_2_ for 10 min. After being washed repeatedly with sterile water, the seeds were placed on moist filter papers for pre-germination at 25 °C in the dark until the radicles appeared.

The AM fungus *Rhizophagus irregularis* Walker & Schuessler (BGC AH01) was provided by the Beijing Academy of Agriculture and Forestry, China. The fungus was propagated by pot culture with *Sorghum bicolor* L. as the host plant in sand for 3–4 months. The mycorrhizal inocula consisted of a mixture of colonized root fragments, spores (68 spores g^−1^), hyphae.

The potting substrate was a mixture of soil and sand (2:1, *w*/*w*). The soil (17 indigenous AM fungal spores g^−1^ soil) was collected from Erdos, Inner Mongolia, China (39°53′ N, 110°1′ E), and passed through a 2 mm sieve. Half of the mixture was sterilized by γ-radiation (dose: 25 kGy, 10 MeV electron beam, China Institute of Atomic Energy, Beijing, China) for sterilization treatment. Previous studies demonstrated that γ-radiation sterilization is a more suitable method of soil sterilization with less impact on soil properties than other methods [62,63]. The unsterilized half was used as the experimental control. The soil had a pH of 8.70 (1:2.5 soil/water), 10.79 g kg^−1^ organic matter, and 4.46 g kg^−1^ available phosphate content (extracted with 0.5 mol L^−1^ NaCO_3_, pH 8.5). Sand was intermittently autoclaved twice at 121 °C for 1 h. Before sowing, the substrates received basal nutrients of 120 mg kg^−1^ N (NH_4_NO_3_), 30 mg kg^−1^ P (KH_2_PO_4_), and 120 mg kg^−1^ K (KH_2_PO_4_ and K_2_SO_4_). For convenience, this mixed substrate was hereinafter referred to as “soil”.

### 4.2. Experimental Procedure

In the inoculation treatment (+M), 800 g of unsterilized (US) or sterilized (S) soil was added to the pots (15 cm in diameter and 14 cm in height), followed by the addition of 40 g of AM fungal inoculum mixed with 400 g of the same soil. For the non-inoculation treatment (−M) under two soil conditions, equivalent autoclaved inoculum (121 °C for 30 min) was added to the pot with 5 mL of AM fungus inoculum filtrate to ensure similar microbiota except for the AM fungus. This procedure could eliminate the problem of differences in microbiotas due to the introduction of exogenous AM fungi between the −M and +M treatments. The filtrate was obtained by passing 10 g of mycorrhizal inoculum in 40 mL of distilled water through a layer of 15–20 μm filter paper. The four treatment groups were as follows: (1) US−M: plants grown in unsterilized soil and not inoculated with AM fungus; (2) US+M: plants grown in unsterilized soil and inoculated with AM fungus; (3) S−M: plants grown in sterilized soil but not inoculated with AM fungus; (4) S+M: plants grown in sterilized soil and inoculated with AM fungus. By comparing the plant performance between S−M and US−M, we can test the hypothesis that soil microbial communities are powerful and indispensable in the soil ecosystem. By comparing the plant performance between S+M and US−M, we could test the hypothesis that AM fungi can efficiently compensate for the loss of indigenous soil microbial communities to maintain plant growth and facilitate secondary metabolite accumulation. Finally, by comparing the plant performance between US+M and S+M/US−M, we could test whether AM inoculation still exerts positive promoting effects on plant growth, even in the presence of an abundant indigenous fungal community in the soil. 

Three seeds were sown in the pot. After 14 days, the seedlings were thinned to two per pot. Plants were watered daily with deionized water to maintain the soil water content to 75% of the field water capacity.

Each treatment included four replicates, and a total of 16 pots with a completely randomized block design were obtained. The experiment was conducted in a controlled environment growth chamber with 700 μmol m^−2^ s^−1^ light intensity, 16 h/8 h (light/dark) photoperiod, 25 °C/20 °C (light/dark) temperature, and 70% relative humidity. No fertilizer or pesticide was applied to the plants during the entire growth period, and all pots received the same management. The plants were harvested after 3 months of growth. Our previous studies revealed that the inoculated plants would have extensive mycorrhizal colonization by this time [28,30].

### 4.3. Net Photosynthetic Rate (Pn) and Stomatal Conductance (Cond) of Plants

Pn and Cond were measured on the third leaf (from the top) by a portable photosynthesis system (Li-6400X, LI-COR Biosciences, Lincoln, NE, USA) 1 day before harvest. The detection parameters were photosynthetic photon flux density of 800 μmol m^−2^ s^−1^, CO_2_ concentration of 400 g m^−3^, and relative humidity of 65% in the leaf chamber. Four replicates of each treatment and two plants per pot were measured randomly in a sunny morning (09:00 AM−11:00 AM).

### 4.4. Plant Growth 

The plant height was measured from the top leaf to the soil surface. The shoots and roots were harvested separately and washed carefully with deionized water repeatedly. Approximately 0.5 g of fresh root samples was used to determine mycorrhizal colonization. Another 0.5 g of roots was stored at −80 °C for RNA extraction and gene expression analysis. The shoot and root dry weights (DWs) were recorded after oven-drying at 60 °C until a constant weight. The dried samples were ground to determine the nutrient, glycyrrhizin, and liquiritin concentrations. 

### 4.5. AM Fungal Colonization

To estimate mycorrhizal colonization, 0.5 g of the root was cut into 1 cm-long segments. The segments were cleaned with 10% KOH, softened with 2% HCl, and stained with 0.05% trypan blue in lactic acid. Thirty stained root segments per pot were placed on slides and observed under a microscope at 100× magnification. Mycorrhizal colonization rate (M%) and arbuscule abundance (A%) were calculated using Mycocalc software [64]. 

### 4.6. Chlorophyll Analysis

Fresh leaves (0.2 g) were cut into pieces and transferred into 20 mL of mixed liquid of acetone and absolute ethyl alcohol (*v:v*, 2:1) in the dark for 24 h until the color of the leaves was completely washed out. The filtering medium was measured at 645 and 663 nm with a UV/vis spectrophotometer (Shimadzu, UV1700, Kyoto, Japan). The chlorophyll concentrations were calculated as follows: Chl a (mg g^−1^) = 12.7 × A_663_ − 2.69 × A_645_; Chl b (mg g^−1^) = 22.9 × A_645_ − 4.68 × A_663_ [65].

### 4.7. Elemental Analysis

The C and N concentrations in the shoots and roots were determined by an elemental analyzer (Vario MAX, Elementar, Germany). The P, K, Ca, and Mg concentrations were measured by ICP-OES (Prodigy, Teledyne Leeman, Hudson, NH, USA) after digestion with HNO_3_ using a Microwave Accelerated Reaction System (Mars 7, CEM Corp, Matthews, NC, USA). Blanks and standard materials (Sulfanilic acid, Sigma-Aldrich, Shanghai, China; GBW10016, China Standard Research Center, Beijing, China) were used to ensure the accuracy of elemental analysis. 

### 4.8. Glycyrrhizin and Liquiritin Analyses

Root ground powder (0.1 g) was weighed accurately and extracted with 15 mL of 67% methanol in an ultrasonic bath (250 W, 40 kHz) for 45 min. Extract solutions were cooled down and filtered through a 0.45 μm filter before detection. Glycyrrhizin and liquiritin were separated and detected by high-performance liquid chromatography (HPLC; Agilent-1200, Santa Clara, CA, USA) through an Agilent ZORBAX-Eclipse XDB-C18 column (250 mm × 4.6 mm, 5 μm). Standard materials of glycyrrhizin (Batch No.110731) and liquiritin (Batch No. 11610) were purchased from the National Institute for the Control of Pharmaceutical and Biological Products, China, to generate the calibration curve [30].

### 4.9. RNA Isolation and Quantitative Real-Time PCR (qRT-PCR) Analysis 

The total root RNA of liquorice was extracted using CTAB (2% CTAB, 2% PVP-40, 0.1 mol L^−1^ Tris-HCl, 0.25 mol L^−1^ EDTA, and 2 mol L^−1^ NaCl), followed by purification with MicroElute RNA Clean-Up Kit (Omega Bio-tek, Norcross, GA, USA) and DNase I (Takara Biotechnology Co. Ltd, Dalian, China). The quantity and quality of the RNA samples were detected by NanoDrop 2000 spectrophotometer (NanoDrop Technologies, Wilmington, DE, USA) and agarose gel electrophoresis. Complementary DNA (cDNA) was synthesized using a RevertAid First Strand cDNA Synthesis Kit (Thermo Fisher Scientific Inc., Wilmington, DE, USA) according to the manufacturer’s instructions. *FPS*, *β-AS*, *CYP88D6*, *CYP72A154*, *LUS*, and *CHS* genes were analyzed using the ABI Prism 7300 Real-Time PCR System (ABI Inc., Carlsbad, CA, USA). qRT-PCR was performed with the same protocols used by Xie et al. [30]. Four independent biological replicates and three technical replicates were analyzed. The relative gene expression level was calculated by the 2^−ΔΔCt^ method and normalized with *β-actin* [66].

### 4.10. Statistical Analysis

All experimental data were tested for normality and homogeneity of variance using the Shapiro–Wilk test and Levene’s test, respectively, prior to statistical analysis. In case of variance homogeneity, data were statistically analyzed using two-way ANOVA, and the significance of differences among treatments was evaluated using Tukey’s HSD test (*p* < 0.05). In the case of heterogeneity of variance, data were analyzed using a nonparametric Kruskal–Wallis test. All data were analyzed by SPSS 19.0 (IBM Corp., Armonk, NY, USA). Data are presented as the mean ± standard error (SE) in the figures and tables. 

## 5. Conclusions

Our results showed that soil indigenous microbial communities play important roles in promoting plant growth. Moreover, AM inoculation could compensate for the loss of soil indigenous microbial communities to support plant growth. Future studies should trace the behavior of the introduced AM fungi in the soil ecosystem and examine the interactions of AM fungi with indigenous microbial communities. The effectiveness of the exogenous and indigenous AM fungi and their interactive effects on plant growth in multiple soil types also need further investigation.

## Figures and Tables

**Table 1:** Mycorrhizal colonization of liquorice plants under different treatments. US and S represent unsterilization and sterilization treatments, respectively. −M and +M represent non-inoculation and AM inoculation, respectively. Data are presented as means ± standard error (SE, *n* = 4). Different lowercase letters after the means indicate a significant difference between corresponding treatments at *p* < 0.05 by Tukey’s HSD test. Mycorrhizal colonization rate (M%) and arbuscule abundance (A%) were analyzed using two-way ANOVA; ns, not significant, * *p* < 0.05, ** *p* < 0.01.

Sterilization (*S*)	Inoculation (*I*)	Mycorrhizal Colonization Rate (M%)	Arbuscule Abundance (A%)
US	−M	31.7 ± 5.2 b	6.0 ± 1.0 bc
	+M	49.9 ± 5.6 a	16.2 ± 3.2 ab
S	−M	0.0 ± 0.0 c	0.0 ± 0.0 c
	+M	65.3 ± 4.0 a	26.6 ± 5.2 a
ANOVA	*S*	*F*_1,12_ = 3.56 ^ns^	*F*_1,12_ = 0.61 ^ns^
	*I*	*F*_1,12_ = 94.07 **	*F*_1,12_ = 36.21 **
	*S* × *I*	*F*_1,12_ = 29.86 **	*F*_1,12_ = 6.69 *

**Table 2:** Height, shoot, and root dry weights of liquorice plants under different treatments. US and S represent unsterilization and sterilization treatments, respectively. −M and +M represent non-inoculation and AM inoculation, respectively. Data are presented as means ± standard error (SE, *n* = 4). Different lowercase letters after the means indicate a significant difference between corresponding treatments at *p* < 0.05 by Tukey’s HSD test. Height, shoot, and root dry weights of liquorice plants were analyzed using two-way ANOVA; ns, not significant, * *p* < 0.05, ** *p* < 0.01.

Sterilization (*S*)	Inoculation (*I*)	Plant Height (cm)	Shoot Dry Weight (g pot^−1^)	Root Dry Weight (g pot^−1^)
US	−M	44.2 ± 2.5 a	1.92 ± 0.10 a	4.31 ± 0.10 a
	+M	47.1 ± 3.0 a	2.50 ± 0.20 a	4.48 ± 0.11 a
S	−M	10.0 ± 0.9 b	0.10 ± 0.02 b	0.23 ± 0.03 b
	+M	49.5± 4.4 a	2.73 ± 0.20 a	4.11 ± 0.40 a
ANOVA	*S*	*F*_1,12_ = 53.445 **	*F*_1,12_ = 120.56 **	*F*_1,12_ = 389.55 **
	*I*	*F*_1,12_ = 80.132 **	*F*_1,12_ = 181.54 **	*F*_1,12_ = 357.90 **
	*S* × *I*	*F*_1,12_ = 64.295 **	*F*_1,12_ = 135.08 **	*F*_1,12_ = 339.84 **

**Table 3:** C, N, P, Ca, K, and Mg concentrations in the shoot and root and C:N:P ratios of liquorice plant under different treatments. US and S represent unsterilization and sterilization treatments, respectively. −M and +M represent non-inoculation and AM inoculation, respectively. Data are presented as means ± standard error (SE, *n* = 4). Different lowercase letters after the means indicate a significant difference between corresponding treatments at *p* < 0.05 by Tukey’s HSD test. C, N, P, Ca, K, and Mg concentrations in the shoot and root and C:N:P ratios of liquorice plant were analyzed using two-way ANOVA; As the exception, root C concentrations and C:N ratios of the treatments were compared by using a nonparametric Kruskal–Wallis test; ns, not significant, * *p* < 0.05, ** *p* < 0.01.

	Sterilization (*S*)	Inoculation (*I*)	C (mg g^−1^)	N (mg g^−1^)	P (mg g^−1^)	K (mg g^−1^)	Ca (mg g^−1^)	Mg (mg g^−1^)	C:N	C:P	N:P
Shoot	US	−M	435 ± 5 a	20.24 ± 0.67 b	1.66 ± 0.09 a	18.53 ± 0.77 b	13.93 ± 0.57 b	2.76 ± 0.16 b	21.55 ± 0.53 a	264 ± 14.71 b	12.2 ± 0.62 b
		+M	435 ± 4 a	19.78 ± 0.49 b	1.60 ± 0.16 a	17.81 ± 1.53 b	11.27 ± 0.35 c	2.34 ± 0.23 b	22.03 ± 0.36 a	280 ± 26.92 b	12.7 ± 1.16 b
	S	−M	411 ± 3 b	24.33 ± 0.58 a	0.42 ± 0.01 b	22.85 ± 0.76 a	25.90 ± 0.75 a	4.48 ± 0.32 a	16.93 ± 0.53 b	962 ± 32.73 a	57.1 ± 3.07 a
		+M	443 ± 3 a	20.41 ± 0.88 b	1.60 ± 0.06 a	16.83 ± 0.60 b	11.96 ± 0.95 bc	2.28 ± 0.21 b	21.85 ± 1.03 a	278 ± 12.31 b	12.7 ± 0.35 b
	ANOVA	*S*	*F*_1,12_ = 4.60 ^ns^	*F*_1,12_ = 12.37 **	*F*_1,12_ = 117.23 **	*F*_1,12_ = 2.89 ^ns^	*F*_1,12_ = 47.97 **	*F*_1,12_ = 12.18 **	*F*_1,12_ = 13.28 **	*F*_1,12_ = 223.70 **	*F*_1,12_ = 947.92 **
		*I*	*F*_1,12_ = 18.09 **	*F*_1,12_ = 10.65 **	*F*_1,12_ = 105.79 **	*F*_1,12_ = 11.67 **	*F*_1,12_ = 104.19 **	*F*_1,12_ = 30.20 **	*F*_1,12_ = 16.72 **	*F*_1,12_ = 206.51 **	*F*_1,12_ = 884.38 **
		*S* × *I*	*F*_1,12_ = 18.01 **	*F*_1,12_ = 6.65 *	*F*_1,12_ = 122.85 **	*F*_1,12_ = 7.23 *	*F*_1,12_ = 34.44 **	*F*_1,12_ = 13.85 **	*F*_1,12_ = 11.32 **	*F*_1,12_ = 227.12 **	*F*_1,12_ = 955.09 **
Root	US	−M	403 ± 10 b	16.26 ± 1.75 b	2.58 ± 0.14 a	7.48 ± 0.15 a	3.83 ± 0.20	0.69 ± 0.07 b	25.53 ± 2.23 a	157 ± 5.20 b	6.24 ± 0.34 b
		+M	413 ± 5 b	16.72 ± 0.82 b	2.43 ± 0.11 a	7.89 ± 0.47 a	4.15 ± 0.35	0.90 ± 0.08 b	24.85 ± 1.16 a	171 ± 9.24 b	6.94 ± 0.57 b
	S	−M	436 ± 2 a	29.64 ± 0.41 a	0.69 ± 0.01 b	4.28 ± 0.05 b	4.40 ± 0.37	1.55 ± 0.06 a	14.71 ± 0.13 b	636 ± 13.23 a	43.4 ± 0.66 a
		+M	422 ± 1b	18.89 ± 0.77 b	2.62 ± 0.15 a	7.95 ± 0.45 a	4.19 ± 0.17	0.85 ± 0.06 b	22.45 ± 0.97 a	153 ± 14.16 b	6.87 ± 0.76 b
	ANOVA	*S*	-	*F*_1,12_ = 53.87 **	*F*_1,12_ = 167.49 **	*F*_1,12_ = 22.00 **	*F*_1,12_ = 1.53 ^ns^	*F*_1,12_ = 40.86 **	-	*F*_1,12_ = 440.14 **	*F*_1,12_ = 47.32 **
		*I*	-	*F*_1,12_ = 23.58 **	*F*_1,12_ = 174.00 **	*F*_1,12_ = 37.24 **	*F*_1,12_ = 0.05 ^ns^	*F*_1,12_ = 14.32 **	-	*F*_1,12_ = 455.94 **	*F*_1,12_ = 37.76 **
		*S* × *I*	-	*F*_1,12_ = 27.98 **	*F*_1,12_ = 208.10 **	*F*_1,12_ = 23.77 **	*F*_1,12_ = 1.11 ^ns^	*F*_1,12_ = 51.36 **	-	*F*_1,12_ = 511.41 **	*F*_1,12_ = 41.40 **
